# Editorial: Rising stars in cancer immunity and immunotherapy 2022

**DOI:** 10.3389/fimmu.2023.1326374

**Published:** 2023-11-07

**Authors:** Cleo Goyvaerts, Christine E. Engeland, Kevin Van der Jeught

**Affiliations:** ^1^ Laboratory for Molecular and Cellular Therapy, Faculty of Medicine and Pharmacy, Vrije Universiteit Brussel, Brussels, Belgium; ^2^ Experimental Hematology and Immunotherapy, Department of Hematology, Hemostaseology, Cellular Therapy and Infectious Diseases, Faculty of Medicine and Leipzig University Hospital, Leipzig, Germany; ^3^ Department of Microbiology and Immunology, University of Miami Miller School of Medicine, Miami, FL, United States; ^4^ Sylvester Comprehensive Cancer Center, University of Miami, Miami, FL, United States

**Keywords:** immunotherapy, microbiome, oncolytic virotherapy, perioperative activity, adoptive cell therapies, personalized medicine, immune checkpoint inhibition

In the late 19^th^ century, William Coley reported over 1,000 partial or even complete cancer regressions showcasing the curative potential of microbial agents (1). A very ‘outside-the-box’ attempt to treat cancer by harnessing the patient’s own immune system (2). Yet, these and similar early cancer immunotherapies were underestimated and even derided for decades. Coining of the cancer immunoediting concept and key discoveries in fundamental immunology, however, enabled a revolution in the treatment of cancer. To illustrate, antiviral and antitumor mRNA-based vaccination was initially considered a daunting approach. The 2023 Nobel Prize in Physiology or Medicine awarded to the mRNA vaccination pioneers Katalin Karikó and Drew Weissman highlights that novel immunotherapeutic modalities are becoming recognized as agents with immense clinical potential. Technological and transdisciplinary advances such as single cell multi-omics and 3D-tissue modeling are further fueling the development of novel strategies encompassing immunostimulatory agents, therapeutic vaccination, adoptive (T) cell therapies, immune checkpoint inhibition (ICI), and more.

Remarkably, 130 years after Coley’s attempts to exploit the cancer patient’s own immune system with bacterial extracts, 4 out of 7 articles of this Research Topic take up his challenge in harnessing microbial agents to treat cancer. In a review article, Goubet reveals the untapped potential of tumor-associated microbes in influencing the development and prognosis of cancer. These microbes provide danger signals and non-self-antigens which make them exploitable stimulating agents that can serve as bull’s-eyes for the immune system, potentially influencing a patient’s response to immunotherapy. Mowat et al. substantiate this view by showing that in colorectal cancer (CRC), bacterial metabolites such as short chain fatty acids (SCFAs) have a Janus-faced role with both protumoral mutagenic and immune-stimulating properties. Importantly, the positive effect of SCFAs on CD8^+^ T cell activation is more pronounced in microsatellite instability high tumors than in chromosomally instable (CIN) CRC, hinting at a mechanism to exploit SCFAs in combination regimens to treat the latter. Turning from bacteria to viruses, Pakola et al. show that an interleukin-2 (IL-2) encoding oncolytic adenovirus improves both survival and tumor control in pancreatic ductal adenocarcinoma (PDAC) in combination with chemotherapy. In their study, the tumor-infiltrating immune cells were activated, with a tumor microenvironment (TME) characterized by chemokine ligand (CCL)-2, CCL-3, CCL-4, interferon (IFN)-γ, and tumor necrosis factor (TNF)-α. In addition, inflammatory cancer-associated fibroblasts (CAFs) made room for antigen-presenting CAFs, owing to reduced IL-6 and immunosuppressive myeloid cell infiltration. Veinalde et al. further highlight the vigor of oncolytic virotherapy for ICI-refractory PDAC. They demonstrate that in PDAC, combined measles virotherapy with anti-Programmed cell Death protein 1 (PD-1) ICI reshapes the TME, induces tumor-specific immunity and significantly prolongs survival. This study also emphasizes the importance of timing in immunotherapy, as the TME remodeling that likely sensitized PDAC to ICI was restricted to a specific time window after virotherapy.

Along these lines, Duerinck et al. advocate that the perioperative period provides a window of opportunity that can be exploited to improve the sobering response rates of glioblastoma patients to ICI. They argue that surgery not only reduces the tumor mass with concomitant depletion of immunosuppressive cells, but also increases tumor antigen uptake and presentation, thereby promoting anti-tumor immunity. Harnessing this window allows local injection of immunotherapies to cover the entire tissue lining of the resection cavity and surpass the drug penetration confines of the blood-brain-barrier upon systemic ICI delivery.

Next to ICI, the advent of cellular immunotherapies is reshaping the preclinical research landscape as well as the treatment paradigms for cancer. Thus, this Research Topic includes two publications focusing on cellular therapies. Ockfen et al. demonstrate that not only processes at the presynaptic side of the immunological synapse, created by cytotoxic effector cells, but also the actin dynamics at the postsynaptic side in cancer cells represent underexplored mechanisms that can be leveraged to improve both recombinant and cellular immunotherapies. Biederstädt et al. review how multiplexed precision gene editing can tackle some of the current challenges that restrain cellular therapies such as limited *in vivo* persistence, functional exhaustion, on-target off-tumor toxicities, and tumor-intrinsic resistance mechanisms.

This Research Topic showcases how a deeper understanding of cancer biology and immunology paired with advanced molecular biotechnology can accelerate the development of novel immunotherapies that leverage innate and adaptive immunity to synergize in fighting cancer. Accordingly, for ICI refractory cancers, such as CIN CRC, PDAC and glioblastoma, novel combination strategies will be key to realize successful immunotherapy. Combination regimens do raise important questions as to selection, timing, and dosing of individual treatment components. Considering patient diversity and tumor heterogeneity, this calls for increasingly personalized approaches. Moving forward, the use of patient-derived material, preferably comprising tumor, immune and stromal cells, should become part of the gold standard workflow in preclinical cancer research. In addition, as long as syngeneic mouse models are irreplaceable for onco-immunological research, preclinical experimental design should include orthotopic, preferably spontaneous tumor models to evaluate combination treatments while bearing in mind clinical regimens. Finally, researchers should contribute to the rapidly expanding repositories of publicly available data and open access publication. Regardless of the strategy used, immunotherapy is progressing to an individualized treatment, calling for continuous efforts to develop clinically standardized methods that provide predictive biomarkers to assist oncologists’ treatment decisions.

We anticipate that the Rising Stars of this Research Topic will continue and inspire others to make daring ‘outside-the-box’ discoveries that can soon earn a place in the revolution of cancer treatment.

## Author contributions

CG: Conceptualization, Validation, Writing – original draft, Writing – review & editing. CE: Conceptualization, Validation, Writing – original draft, Writing – review & editing. KJ: Conceptualization, Validation, Writing – original draft, Writing – review & editing.
